# Surgical treatment of pelvic organ prolapse using colpocleisis: A case series

**DOI:** 10.12669/pjms.39.5.7600

**Published:** 2023

**Authors:** Hassan M Elbiss, Omaema Al-Baghdadi

**Affiliations:** 1 Hassan M Elbiss, MD, MRCOG, FRCOG Departments of Obstetrics and Gynaecology, College of Medicine and Health Sciences, UAE University, Al Ain, UAE; 2 Omaema Al-Baghdadi, MRCOG, CCT, College of Medicine and Health Sciences, UAE University, Al Ain, UAE

**Keywords:** Colpocleisis, Female urinary incontinence, Pelvic organ prolapse

## Abstract

**Objective:**

Pelvic organ prolapse (POP) is a common condition. With increasing lifespan and emphasis on quality of life worldwide, older women with POP may prefer surgical treatment, including colpocleisis. We reviewed the outcome of POP in a case series of colpocleisis.

**Methods:**

This study was conducted between 2006 and 2011. Patients had confirmed POP on examination and underwent partial and total colpocleisis. We compared patients’ demographic characteristics, POP severity and surgical outcomes.

**Results:**

In total, 55 patients were included. The patients were aged 78.9 + 6.7 years and had body mass index (BMI) of 26.9 ± 4.2. Among the total, 44 (80%) and 11 patients (20%) underwent partial and total colpocleisis respectively. Patients undergoing partial colpocleisis had procidentia less often than those undergoing total colpocleisis (18% v 64%, p=0.01). Mean blood loss and operative time were 157.6 ml ± 119.0 and 65.1 ± 20.3 minutes respectively. Partial colpocleisis had less blood loss (mean 135.7 v 227.2 ml, p=0.02) and shorter operative time (mean 62.6 v 75.0 min, p=0.18).

**Conclusion:**

Partial compared to total colpocleisis was associated with shorter operative time and less blood loss. Colpocleisis is a suitable surgical treatment for POP in elderly women who do not wish to preserve the vagina for sexual intercourse.

## INTRODUCTION

Pelvic organ prolapse (POP), a common uro-gynaecological condition, is prevalent among elderly women^1^ and affects the quality of life to a significant extent.^2^-^4^ Some factors that may predispose women to develop POP are age, weight, vaginal delivery and multiparity.^4^,^5^ The prevalence of POP is expected to increase in the coming years and so is the need for its surgery.^6^ The incidence of POP is reported to be nearly 50%.^7^ Common surgical options for managing POP in elderly women include obliterative and reconstructive procedures.^8^,^9^ The choice of surgery depends upon the patient’s goals, health status and desired outcomes. Colpocleisis, an obliterative surgical procedure, involves surgical closure of vagina to fix the prolapse and is recommended for women who are no longer sexually active.^10^-^12^ Colpocleisis has two types, partial and total colpocleisis. This surgery can be performed either with the local anesthesia or general anesthesia.^13^,^14^

It has been demonstrated that the likelihood of women undergoing surgery for POP during their lifetime is high and therefore strong evidence is needed for available surgical options for managing POP, including colpocleisis.^1^,^15^ A previous European study conducted in women who underwent colpocleisis showed that colpocleisis is a potentially useful treatment for POP. There was no recurrence of prolapse in these women and colpocleisis had a positive impact on quality of life but urinary symptoms such as urinary tract infections (UTIs) following colpocleisis were a major concern.^16^ In this study, the sample size was small and had a risk of bias due to retrospective nature of the study. Another retrospective cohort study in Korea, evaluating outcomes of partial colpocleisis procedure for managing advanced apical prolapse showed a high success rate and low regret rate among elderly women who do not wish to continue sexual intercourse.^17^ However, there were no comparative descriptive studies of partial versus total colpocleisis.

To our knowledge, there is limited evidence available related to colpocleisis procedure. The objective of this study was to review the procedure and its outcomes in women with POP, comparing partial with total colpocleisis. We aimed to compare patients’ demographic characteristics, POP severity and surgical outcomes.

## METHODS

This study was conducted between 2006 and 2011 in Peterborough District Hospital, UK, approved by the hospital’s audit department. Patients’ data were used with their permission in an annonymised fashion.

### Setting and participants

Physical examinations were conducted to confirm POP, stage of prolapse, and the compartments involved; all examinations were performed using the International Continence Society Pelvic Organ Prolapse Quantification (POP-Q) staging system. Patients who opted for surgical treatment were scheduled for surgery.

### Surgical procedure

Thorough counseling was provided regarding reconstructive and obliterative treatment options, including a discussion of the potential benefits and risks of both procedures. Preoperative urodynamics studies were performed if indicated. During the preoperative assessment, each patient underwent a comprehensive evaluation of the variables. A total colpocleisis referred to the removal of the majority of the vaginal epithelium from within the hymenal ring posteriorly, and to within 0.5-2.0 cm of the external urethral meatus anteriorly. A partial colpocleisis referred to the technique of leaving some portion of the vaginal epithelium in place, providing drainage tracts for cervical or other upper genital discharge in accordance with the technique of LeFort.

The procedures were performed according to the following technique (Fig.1): A rectangle of vaginal mucosa was marked and removed from the posterior vaginal wall. The Foley catheter balloon was outlined at the level of the bladder neck. A rectangle of the vaginal mucosa was marked and was removed from the anterior vaginal wall. The posterior vaginal wall was incised with a scalpel. The surgeon dissected the posterior vaginal wall epithelium off the underlying muscularis using Metzenbaum scissors and traction with Allis clamps. The surgeon’s index finger was used to provide exposure of the tissue plane to be dissected. The posterior vaginal epithelium was dissected off the underlying muscularis, leaving a flap of tissue available to assist with packing the vaginal wall while attention was turned to the anterior side of the vagina.

**Figure:**
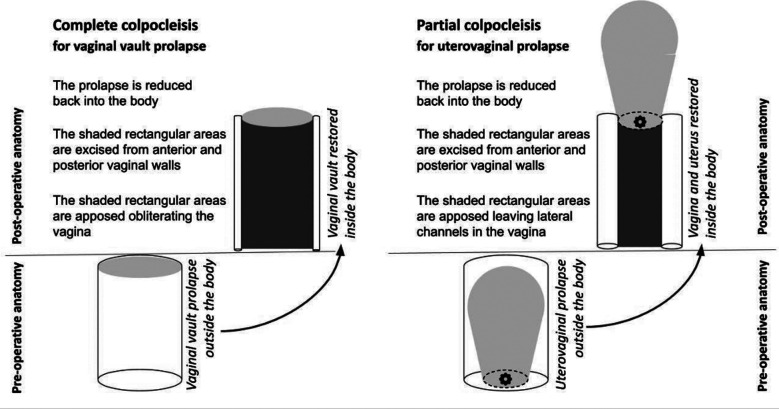
Fig.1:

A vaginal laparotomy sponge was placed between the flap and the posterior vaginal wall muscularis for hemostasis. The flap was secured to the posterior vaginal wall with Allis clamps. The anterior vaginal epithelium was removed. The epithelial corners of the anterior and posterior rectangles were sutured together over the cervix for hemostasis. The sutures were cut long and tagged with Kelly clamps to facilitate manipulation of the vagina for surgical exposure. A row of interrupted sutures brought the cut edges of the anterior and posterior vaginal epithelium together over the cervix. The anterior and posterior muscularis layers were imbricated over the cervix using absorbable suture, creating a septum of tissue. After a series of imbricating sutures of the muscularis and formation of bilateral tunnels of vaginal mucosa, the prolapse was reduced. The mucosal edges were approximated with interrupted 2-0 polyglactin 910 sutures.

### Variables and data sources

Demographic data and symptoms of prolapse were collected during each patient’s first visit to a participating gynaecology unit. Included patients-reported symptoms of prolapse; they also underwent prolapse assessment using POP-Q staging. Information regarding the use of pessaries (ring or shelf) was collected. Outcome measures included estimated blood loss, operative time, hospital stay and catheter removal.

### Statistical methods

We compared patient demographic characteristics, POP severity, and surgical outcomes between the two groups, i.e. partial and total colpocleisis surgery. Descriptive statistics were used to summarize demographic and patient’s characteristics. Continuous variables were expressed as mean (standard deviation) and t-test was used to analyze this data. To express categorical data, numbers and percentages were used and Fisher exact test was used to analyze it. The data was analyzed using Statistical Package for Social Sciences (SPSS) version 16. We considered the results statistically significant if p value was found to be less than 0.05.

## RESULTS

There were 60 patients in our case series during the study period. Data on five patients were missing concerning a number of variables (<10% loss of data). Thus data on 55 patients were analyzed.

### Patient characteristics

The included 55 patients had confirmed POP on examination and underwent colpocleisis, either total or partial. Overall patients were aged 78.9 ± 6.7 years and had BMI of 26.9 ± 4.2. Among the total, 44 (80%) and 11 patients (20%) underwent partial and total colpocleisis respectively. A comparison of the patients’ characteristics according to the type of surgery is given in Table-I. Patients undergoing partial colpocleisis had procidentia less often than those undergoing total colpocleisis (18% v 64%, p=0.01).

**Table I:** Baseline characteristics of patients undergoing colpocleisis.

Feature	Partial colpocleisis (n=44)	Total colpocleisis (n=11)	p-value
Age (years) +	78.3 (6.4)	81.3 (7.3)	0.23
Body mass index +	27.1 (4.5)*	26.3 (2.5)**	0.45
Hormone Replacement Therapy	15 (58%)	3 (43%)	0.96
Grade III Vault prolapse	7 (16%)	2 (18%)	0.99
Procidentia	8 (18%)	7 (64%)	0.01
Grade III Cystocele	11 (25%)	1 (9%)	0.48
Grade III Rectocele	7 (16%)	0 (0%)	0.37
***Pessaries (Ring or Shelf)***			
Ring	10 (23%)	2 (18%)	0.35
Shelf	16 (36%)	5 (45%)
Both	5 (11%)	3 (28%)	
None/Others	13 (30%)	1 (9%)	
Vaginal Hysterectomy	12 (27%)	2 (18%)	0.84
Anterior posterior repair	7 (16%)	1 (9%)	0.98

+Mean (SD);

* missing=2;

** missing=1.

### Outcomes

The patients’ outcomes according to the type of surgery. Overall mean blood loss and operative time was 157.6 ml ± 119.0 and 65.1 ± 20.3 minutes respectively. Partial colpocleisis had less blood loss (mean 135.7 v 227.2 ml, p=0.02) and shorter operative time (mean 62.6 v 75.0 min, p=0.18) (Table-II).

**Table-II:** Outcomes of patients undergoing colpocleisis.

Feature	Partial colpocleisis (n=44)	Total colpocleisis (n=11)	p-value
Estimated blood loss (ml) +	135.7 (85.01)#	227.2 (179.3)	0.02
Operation time (min) +	62.6 (19.2)*	75.0 (22.6)**	0.18
***Hospital stay (days)##***			
≤3	37 (88%)	8 (89%)	0.08
>3	5 (12%)	1 (11%)	
***Catheter removed$***			
Day 1	33 (0.82)	10 (0.91)	0.88
Day 2	4 (0.09)	0	
Kept	4 (0.09)	1 (0.09)	

+Mean (SD);

* missing=13;

** missing=3; #missing=9; ##missing=2 per group; $missing=4 from partial group.

## DISCUSSION

This study provided comparative analysis of two types of colpocleisis, partial versus total. Three-fourth of the patients underwent partial colpocleisis surgery and only one-fourth underwent total colpocleisis surgery. Procidentia was statistically more common among the patients who underwent total colpocleisis surgery. Less blood loss was observed in partial colpocleisis surgery as compared to the total colpocleisis surgery. To our knowledge, this is one of the largest case series concerning colpocleisis surgery giving comparative information concerning partial and complete procedures for the first time.

Interpretation of our findings should be in light of previous studies. A retrospective study conducted in colpocleisis patients confirmed that colpocleisis is a useful treatment for pelvic organ prolapse.^16^ Our study supports this finding. Another retrospective study showed positive outcomes of partial colpocleisis surgery in women who were no longer engaged in sexual intercourse.^17^ Our study is in line with this observation. Colpocleisis provides good relief of pelvic floor symptoms without significant morbidity.^18^ A previous study showed significant effects of resconstructive surgery on sexual function in women with POP and/or stress incontinence.^3^

In our study, we included only elderly women and hence sexual function was not a major concern for this population. The morbidity and mortality associated with the surgery is largely related to the health status of this elderly segment of the population.^19^,^20^ Stress incontinence can be addressed with a concomitant procedure such as a mid-urethral synthetic sling.^21^ A previous study has shown that the likelihood of women undergoing surgery for POP is high.^15^ Our study provides comparative evidence concerning a surgical option to manage this prevalent condition. Colpocleisis surgery, both total and partial, are useful surgical options for managing POP.^22^,^23^ Urogynaecologists should consider this option while treating POP in older women who do not wish to engage in sexual intercourse.

### Strength and Limitations of the study

The strength of our study was that it was reported according to the guidelines mentioned in STROBE checklist.^24^ All of the data that was missing were transparently reported with their numbers in footnotes of the tables. In any case, the patient data loss was small in proportion. The sample size of our study was small and it would be difficult to generalize these results. One perceived limitation of this study may be that we did not perform quality of life assessment following colpocleisis surgery, something that should be addressed in future research.

## CONCLUSION

Colpocleisis is a useful surgical treatment for POP in elderly women who do not wish to preserve the vagina for sexual intercourse. In our study, partial compared to total colpocleisis was associated with shorter operative time and less blood loss. As the population ages, these procedures stand to become increasingly popular treatment options.

### Authors’ Contribution:

**HME:** Conceived, designed, did the statistical analysis, data collection and editing of the manuscript. Responsible and accountable for the accuracy or integrity of the work.

**OAB:** Assisted in the analysis, manuscript writing, editing and formatting
